# Instantaneous phase difference analysis between thoracic and abdominal movement signals based on complementary ensemble empirical mode decomposition

**DOI:** 10.1186/s12938-016-0233-7

**Published:** 2016-10-06

**Authors:** Ya-Chen Chen, Tzu-Chien Hsiao

**Affiliations:** 1 Institute of Computer Science and Engineering, National Chiao Tung University, Hsinchu, 30010 Taiwan; 2 Department of Computer Science, National Chiao Tung University, Hsinchu, 30010 Taiwan; 3 Institute of Biomedical Engineering, National Chiao Tung University, Hsinchu, 30010 Taiwan; 4 Biomedical Electronics Translational Research Center and Biomimetic Systems Research Center, National Chiao Tung University, Hsinchu, 30010 Taiwan

**Keywords:** Instantaneous phase difference, Lissajous figure, Complementary ensemble empirical mode decomposition, Thoracoabdominal motion

## Abstract

**Background:**

Thoracoabdominal asynchrony is often adopted to discriminate respiratory diseases in clinics. Conventionally, Lissajous figure analysis is the most frequently used estimation of the phase difference in thoracoabdominal asynchrony. However, the temporal resolution of the produced results is low and the estimation error increases when the signals are not sinusoidal. Other previous studies have reported time-domain procedures with the use of band-pass filters for phase-angle estimation. Nevertheless, the band-pass filters need calibration for phase delay elimination.

**Methods:**

To improve the estimation, we propose a novel method (named as instantaneous phase difference) that is based on complementary ensemble empirical mode decomposition for estimating the instantaneous phase relation between measured thoracic wall movement and abdominal wall movement. To validate the proposed method, experiments on simulated time series and human-subject respiratory data with two breathing types (i.e., thoracic breathing and abdominal breathing) were conducted. Latest version of Lissajous figure analysis and automatic phase estimation procedure were compared.

**Results:**

The simulation results show that the standard deviations of the proposed method were lower than those of two other conventional methods. The proposed method performed more accurately than the two conventional methods. For the human-subject respiratory data, the results of the proposed method are in line with those in the literature, and the correlation analysis result reveals that they were positively correlated with the results generated by the two conventional methods. Furthermore, the standard deviation of the proposed method was also the smallest.

**Conclusions:**

To summarize, this study proposes a novel method for estimating instantaneous phase differences. According to the findings from both the simulation and human-subject data, our approach was demonstrated to be effective. The method offers the following advantages: (1) improves the temporal resolution, (2) does not introduce a phase delay, (3) works with non-sinusoidal signals, (4) provides quantitative phase estimation without estimating the embedded frequency of breathing signals, and (5) works without calibrated measurements. The results demonstrate a higher temporal resolution of the phase difference estimation for the evaluation of thoracoabdominal asynchrony.

## Background

### The clinical background of phase difference estimation in breathing

Breathing is one of the most essential processes for sustaining human life [1, 2]. In the quiet tidal breathing of a healthy human, the thoracic wall movement (TWM) and abdominal wall movement (AWM) occur nearly simultaneously and move synchronously (i.e., phase difference between 0° and 20°) [3–9]. The asynchrony (i.e., a phase difference) between the TWM and AWM has been found under different breathing conditions. For instance, various asynchronies have been found in people engaging in breathing exercise [7, 10]. In clinical practice, thoracoabdominal asynchrony has been measured to determine the underlying mechanism of patients’ respiratory system and to diagnose respiratory diseases [3, 4, 11]. Estimating thoracoabdominal asynchrony presents information about the time shift of AWM or other respiratory movements to the TWM. This shift demonstrates the disability of the respiratory mechanism. Specially, the estimation of asynchrony can be used to assess the function of the respiratory system. Thoracoabdominal asynchrony has been found in patients with chronic obstructive pulmonary disease [4], diaphragmatic weakness [12, 13], and upper airway obstruction [9], as well as after cardiothoracic surgery [3]. In addition, the “respiratory rate variability” (in analogy to heart rate variability or pulse rate variability) has not been established yet since it is difficult to determine the instantaneous respiratory rate unambiguously [14]. It is difficult because most of the measurements of thoracoabdominal asynchrony need to segment respiratory signal into breath-by-breath [15]. As the recent advances in the field of the pulse rate variability research have demonstrated that the “instantaneous pulse rate variability” improved the temporal resolution and also helped in frequency exploration of cardiovascular autoregulation (e.g., autonomic nervous system function) [16], it was thought that the development of the instantaneous respiratory pattern could also be helpful for exploring how the breathing modulates autonomic nervous system.

### The signal processing required for phase difference estimation

To estimate the phase relation of thoracoabdominal motions (i.e., TWM and AWM signals), various approaches that entail using respiratory inductance plethysmography (RIP) have been proposed [3, 17]. On the research path of thoracoabdominal motions, the Lissajous figure analysis (also called loop analysis) technique is a conventional method for estimating the phase difference between TWM and AWM signals. The technique, which has been widely used in clinical applications [3, 18, 19] and research [20, 21], first assumes that both the TWM and AWM signals are sinusoidal [20]. For calculating the phase difference, first, a filter is applied to these signals for noise reduction. Second, when the relation of these signals is drawn into an XY plot, in which the x-axis represents AWM and the y-axis represents TWM, a loop can be found. Finally, analyzing the loop then produces one value of a phase difference for each cycle of breathing. This temporal resolution (i.e., only a single value for each breath) of the phase analysis leaves room for improvement. Another problem is that the TWM and AWM signals are non-sinusoidal patterns in most cases, and estimation errors may increase when the signals deviate from being sinusoidal [22, 23]. To enhance the performance of phase-angle calculation in various respiratory patterns and to improve the temporal resolution of the estimation, a cross-correlation method was used for phase analysis [23]. In this method, both the TWM and AWM signals are first partitioned into segments. The optimal time-shift of each segment is then searched for estimating the phase difference between the TWM and AWM signals. The breath-by-breath segmentation and search of the optimal time-shift enable the intensive computation of phase difference calculation [15]. Other previous studies have reported time-domain procedures with the use of band-pass filters for analyzing asynchrony [15, 24]. The difficulties among all conventional approaches are that the thoracoabdominal motions measured using RIP have time-varying amplitudes and frequencies, and the measurements are easily impeded by noises such as movement artifacts (low-frequency, high-amplitude noise) and cardiogenic oscillations (high-frequency, low-amplitude noise). Thus, filtering noise from the original signal or reducing the sampling rate are necessary to increase the accuracy of phase-angle estimation [23]. Nevertheless, the band-pass filter, which is used to extract the original respiratory signals, induces a phase delay during phase-angle estimation, as mentioned in [15] (i.e., “the order of the linear-phase band-pass FIR filter was set to 200, corresponding to a 2-s time delay”). To prevent such a delay, a zero-phase filter which processes the input data in both the forward and reverse directions can be used. The other studies [25, 26] demonstrated that an empirical mode decomposition (EMD)-based filter can adaptively filter noises without any phase delay. Recently, Chen et al. [21] demonstrated a combination approach of complementary ensemble empirical mode decomposition (CEEMD) and Lissajous figure analysis for analyzing the phase difference of thoracoabdominal motions measured using RIP. The results of their 29-human-subject experiment indicated a significant difference in the phase difference between thoracic breathing (TB) and abdominal breathing (AB). However, the temporal resolution of the phase analysis result was still limited to being per breathing cycle.

### The aim and the organization of the current study

In this paper, we present a novel procedure for estimating the instantaneous phase difference (IPD) of RIP signals between TWM and AWM. This procedure offers five advantages: (1) improves the temporal resolution, (2) does not introduce a phase delay, (3) works with non-sinusoidal signals, (4) provides quantitative phase estimation without estimating the embedded frequency of breathing signals, and (5) works without calibrated measurements. Experiments on two simulated time series (i.e., sinusoidal and triangular signals [23]) and human-subject respiratory data were conducted for validation. In addition to the proposed IPD method, the newer version of loop analysis reported in [21] and the automatic phase estimation procedure (APEP) invented in [15] were applied to the data for comparison. Throughout this paper, the TWM signal measured using an RIP sensor is denoted as RIP_TWM_, and the AWM signal measured using the RIP sensor is denoted as RIP_AWM_. The mathematical symbol “±” is used to denote mean ± standard deviation.

## Methods

### A novel method for instantaneous phase difference estimation

To estimate the IPD from RIP_TWM_ and RIP_AWM_ signals, a novel method for IPD estimation was used as follows:

1. CEEMD was first used to decompose original RIP signals into intrinsic mode functions (IMFs) [27].
2. The main components of the RIP signals were then selected from the IMFs [28]; they are the main component of the RIP_TWM_ signal and the main component of the RIP_AWM_ signal.
3. Finally, the IPD was computed according to the instantaneous phase of the main component extracted from the RIP_TWM_ and instantaneous phase of the main component extracted from the RIP_AWM_ signal.

#### Signal decomposition using CEEMD

CEEMD is a method derived from EMD [29], which is used to decompose biosignals into oscillatory modes (called IMFs) with different frequencies without introducing any phase delay [30]. Through EMD, a signal *x*(*t*) can be decomposed into IMFs and a final residue as follows:

1 $$x\left( t \right) = \mathop \sum \limits_{i = 1}^{A} IMF_{i} \left( t \right) + r(t)$$

where *IMF*_*i*_(*t*) represents the IMF, the *i* goes from *1* to *A*, *r*(*t*) is the residue of the raw data *x*(*t*), and *t* represents the time in seconds. The original signal is decomposed into sequential IMFs by using an EMD-based method. The EMD-based method is an iterative process and can be stopped whenever the goal of a user is met. For the proposed IPD method, one can have more or less IMFs extracted from an original as long as the main components are extracted in order to calculate the phase difference of the TWM and AWM signals. In this study, the *K* was chosen to be 10. Notably, the maximum number of the IMFs that can be extracted from a signal is limited by the sampling rate. The EMD process will be stopped when no more IMFs can be extracted from a signal.

The IMFs are the signals extracted from the original signal by using EMD; they satisfy two conditions [29] so that they can be used to calculate the instantaneous phase without introducing unwanted fluctuations induced by asymmetric wave forms. The final residue is the remainder of the original signal after the extraction of all the IMFs. The first condition requires that the number of extrema and the number of zero crossings in the data set either equal or differ at most by one. The second condition is that the mean value of the envelope defined by the local maxima and the envelope defined by the local minima is zero at any point. The first condition is similar to the traditional narrow band requirements for a stationary Gaussian process. The second condition modifies the classical global requirement to a local one. In the EMD procedure, the frequency corresponding to the activities of the IMF obtained (i.e., 1st IMF, 2nd IMF, …, 10th IMF) becomes increasingly lower. This is because during the procedure, the IMFs are sequentially extracted from the original signal. The EMD-based method iteratively extracts the mean values of the envelope defined by the local maxima and local minima to produce an IMF. This process stops when the original becomes a monotonic function from which no more IMF can be extracted. The frequencies decrease with the increasing number of the IMF. For the final IMF, the frequency descends approximately to zero. The number of IMFs that can be extracted from a signal is limited by the sampling rate of the signal.

**Fig. 1:**
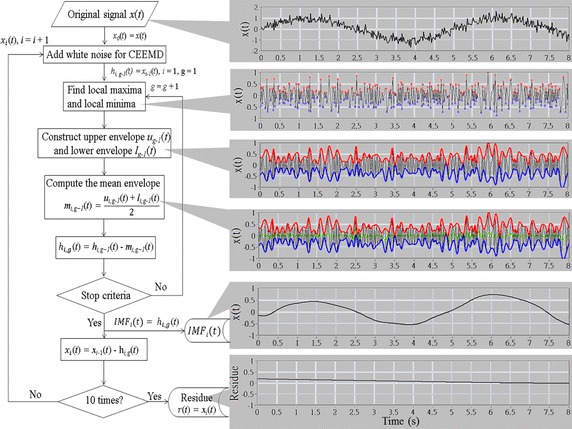
An illustration to the procedure of CEEMD. The *left part* of the figure shows the flow chart of CEEMD and the *right part* shows the instance of sample signals, sample envelope signals (*red line* for upper envelope, *blue line* for lower envelope, and *green line* for mean envelope), and the sample of the extracted signals

EMD consists of an inner loop that extracts an IMF from an input signal and an outer loop that subtracts the obtained IMF from the original signal and then submits the remaining signal into the inner loop for obtaining the next IMF (see Fig. 1). The procedure of the outer loop is as follows:

1. The original signal *x(t)* is submitted into the inner loop to obtain an IMF.
2. After an IMF is generated through the inner loop, the IMF is subtracted from the original signal (i.e., $x_{i} \left( t \right) = x_{i - 1} \left( t \right) - IMF_{i} \left( t \right)$).
3. The inner loop is restarted to obtain the next IMF according to the resulting signal *x*_*i*_ *(t)*.

EMD stops when no more IMFs can be extracted from a signal. The procedure of the inner loop is shown as follows:

1. The local maxima and minima are detected in the input signal.
2. The local maxima (all the peaks of the signal) and minima (all the valleys of the signal) are connected using cubic spline interpolation as the upper (i.e., *u*_*i, g*−*1*_ *(t)*) and lower (i.e., *l*_*i, g*–*1*_ *(t)*) envelopes, respectively.
3. The mean envelope (i.e., *m*_*i, g*−*1*_ *(t)*) is calculated as the mean value of the upper and lower envelopes.
4. The mean envelope is subtracted from the input signal (i.e., $\it \it {\text{h}}_{i,\,g} \left( t \right) \, = \, h_{i,\,g - 1} \left( t \right) \, - {\text{m}}_{i,\,g - 1} (t)$) and the procedure is repeated from the first step.

The inner loop iterates this procedure until the mean envelope approaches zero. Specifically, the inner loop is stopped on the basis of evaluation function $\delta \left( t \right) = {{\left( {u_{i,\,g - 1} \left( t \right) + l_{i,\,g - 1} \left( t \right)} \right)} \mathord{\left/ {\vphantom {{\left( {u_{i,\,g - 1} \left( t \right) + l_{i,\,g - 1} \left( t \right)} \right)} {\left( {u_{i,\,g - 1} \left( t \right) - l_{i,\,g - 1} \left( t \right)} \right)}}} \right. \kern-0pt} {\left( {u_{i,\,g - 1} \left( t \right) - l_{i,\,g - 1} \left( t \right)} \right)}}$. The inner loop is iterated until δ(t) < 0.2 for 95 % of the total length of δ(t), while δ(t) < 2 for the remaining 5 %. Additional details related to the stopping criteria of EMD-based methods are in [31]. The final subtraction result is denoted as an IMF.

CEEMD is an extension of EMD, which was proposed for solving the mode mixing problem and boundary effect problem of EMD [27]. The mode mixing problem is defined as a pattern of signals whose activities reside within the same frequency of different IMFs. When that happens, the IMFs extracted may not be monocomponent. This occurs when EMD fails to extract the activities within the same frequency to become a single IMF. CEEMD processes numerous instances of positive white noise and negative white noise to the signal during the extraction of IMFs. According to the literature [27, 32], adding white noise provides a uniform reference scale distribution and enhances EMD to prevent mode mixing. The boundary effect problem indicates that when generating the upper and lower envelopes during the EMD procedure, EMD uses zero as the amplitude of the envelope at the start and end of the signal. This manner of defining the values of the boundaries (i.e., the start and end of a signal) engenders an undesired pattern of the generated IMFs. CEEMD processes uniformly random values of boundaries. This was proved to prevent undesirable patterns in the boundaries of the extracted IMFs. The concept of CEEMD in decreasing the effect of mode mixing in EMD was applied by adding white noise [27] to the signal as follows:

2 $$\left[ {\begin{array}{*{20}c} {S_{ + } (t)} \\ {S_{ - } (t)} \\ \end{array} } \right] = \left[ {\begin{array}{*{20}c} 1 & 1 \\ 1 & { - 1} \\ \end{array} } \right]\left[ {\begin{array}{*{20}c} {x(t)} \\ {w(t)} \\ \end{array} } \right]$$

where *x*(*t*) represents the raw data of the signal, *w*(*t*) denotes white noise, *S*_+_(*t*) is the mixture of the raw data adding positive white noise, and *S*_−_(*t*) is the mixture of the raw data adding negative white noise. The final IMF is the ensemble of IMF with both positive and negative noises. Figure 1 illustrates the CEEMD procedure. We added 50 instances of positive Gaussian white noise and 50 instances of negative Gaussian white noise. The white noise level was set to 25 % of the standard deviation of the respiratory signals.

#### Main component extraction

After the original signal was decomposed into IMFs, the main component of the RIP signal was determined according to the rule proposed in [28], in that the IMF with the highest energy density is the main component of the RIP signal. The energy density of an IMF is calculated as follows:

3 $$\bar{E}_{i} = \frac{1}{K}\int\limits_{t = 1}^{K} {IMF_{i}^{2} (t)_{{}} dt}$$

where $\bar{E}_{i}$ is the energy density of the *i*th IMF and *K* is the total duration of *IMF*_*i*_(*t*). The frequency of an IMF is calculated as follows:

4 $$\bar{T}_{i} = \frac{{\mathop \sum \nolimits_{j = 1}^{a - 1} \left( {P_{i} \left[ {j + 1} \right] - P_{i} \left[ j \right]} \right)}}{a - 1}$$

This equation was used to calculate the average period of the breathing cycles of the *i*th IMF by averaging all the periods determined according to the peaks of the *i*th IMF. *P*_*i*_[*j*] is the sample time of the *j*th peak of *IMF*_*i*_(*t*). The term $\bar{T}_{i}$ represents the average period of the *i*th IMF, where *a* is the total peak number. The average frequency of an IMF was calculated as the inverse of its average period given by:

5 $$\bar{f}_{i} = \frac{1}{{\overline{{T_{i} }} }}$$

#### Instantaneous phase difference calculation

The instantaneous phases of a main component signal are calculated using normalized direct quadrature in the following equation [33]:

6 $$\theta_{i}^{{}} (t) = \tan^{ - 1} \frac{{\sqrt {1 - {\text{N}}IMF_{i}^{2} (t)} }}{{{\text{N}}IMF_{i} (t)}}$$

where N*IMF*_i_(*t*) represents the normalized *i*th IMF. The IPD (*θ*_*IPD*_) between the instantaneous phase of AWM (*θ*_*AWM*_) and the instantaneous phase of TWM (*θ*_*TWM*_) is then calculated as follows:

7 $$\theta_{IPD} = \left| {\theta_{AWM} - \theta_{TWM} } \right|$$

The *i*th IMF can be normalized to N*IMF*_i_(*t*) through the following procedure [33]:

1. $\left| {IMF_{{{\text{i}},{\text{k}}}} \left( t \right)} \right|$ is obtained for k = 0 by assigning $\left| {IMF_{\text{i}} \left( t \right)} \right|$ to $\left| {IMF_{{{\text{i}},{\text{k}}}} \left( t \right)} \right|$.
2. All the local maxima of $\left| {IMF_{{{\text{i}},{\text{k}}}} \left( t \right)} \right|$ are identified.
3. All the maxima points are connected using cubic spline interpolation to generate the empirical envelope of $\left| {IMF_{{{\text{i}},{\text{k}}}} \left( t \right)} \right|$ as *e*_k_(t).
4. *IMF*_i, k_(*t*)/*e*_k_(t) is assigned to $\left| {IMF_{{{\text{i}},{\text{k + 1}}}} \left( t \right)} \right|$, and *e*_k_(t) is used to pointwise normalize *IMF*_i, k_(*t*).
5. The procedure is repeated from step 1.

When all the $\left| {IMF_{{{\text{i}},{\text{k + 1}}}} \left( t \right)} \right|$ values in step 5 are less than or equal to [−1, 1], the normalization is complete. $\left| {IMF_{{{\text{i}},{\text{k + 1}}}} \left( t \right)} \right|$ is then assigned to N*IMF*_i_(*t*) as the final result.

### Experiment on simulated time series

The current study tested two types of signals (sinusoidal vs. triangular) × 6 degree levels (20°, 30°, 50°, 90°, 140°, and 170°) × 2 setups of noise (correlated noise vs. uncorrelated noise) as the simulated time series, resulting in 24 simulation setups.

#### Sinusoidal signals

For the first simulation, sinusoidal signals *s*_1_(*t*) and *s*_*2*_(*t*) were respectively used to simulate pure TWM and AWM signals as follows:

8 $$s_{1} (t) = A\sin \left( {\frac{2\pi }{T} - t} \right)$$

9 $$s_{2} (t) = A\sin \left( {\frac{2\pi }{T}t + \theta_{0} } \right)$$

where A is the amplitude (A = 1 in this study), *T* is the cycle period (*T* = 5 s in this study), and *θ*_0_ is the initial phase. In this simulation, *θ*_0_ = 20°, 30°, 50°, 90°, 140°, and 170° [3, 9, 13] were tested. The value *T* = 5 s was used because the normal range of the respiratory rate of adults is 12 (0.2 Hz) to 18 (0.3 Hz) breaths per minute [34–36].

#### Triangular signals

**Fig. 2:**
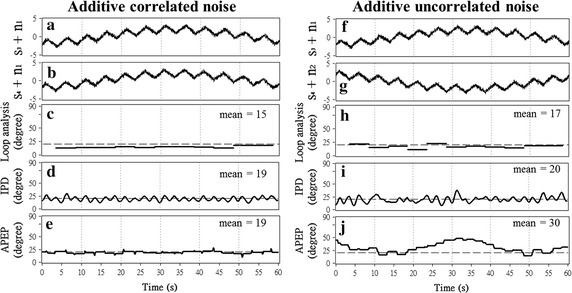
Sample result of the phase difference estimations on simulated signals. A 60-s sample result of phase difference estimations on two triangular signals *s*_3_(*t*) and *s*_*4*_(*t*) with correlated (**a**, **b**) and uncorrelated (**f**, **g**) noises and phase difference *θ*_0_ = 20° done by using the loop analysis (**c**, **h**), IPD method (**d**, **i**), and APEP (**e**, **j**). The frequency of *s*_3_(*t*) and *s*_*4*_(*t*) was set to 0.2 Hz. The *dashed line* represent the gold standard (i.e., *θ*_0_ = 20°). The absent of data for loop analysis in 0–2.5 and 58.5–60 s was caused by the incompleteness of cycles. The loop analysis in fact only generates one output value per cycle of breathing at the end of each cycle. The* straight lines* drawn in the* panel* **c** throughout the beginning to the end of each cycle are manually produced after the analysis. Loop analysis only has one value per cycle in the end of cycles. The phase differences of the cycles are filled with that output value manually in order to make the figure more comprehensive

Triangular waves, *s*_3_(*t*) and *s*_4_(*t*), were also respectively used to simulate TWM and AWM (see Fig. 2). The formula for *s*_3_(*t*) is shown as follows:

10 $$s_{3} (t) = \left\{ {\begin{array}{*{20}r} \hfill {\frac{4A}{T}t,} & \hfill \quad {0 \le (t - mT) \le \frac{T}{4}} \\ \hfill { - \frac{4A}{T}t + 2A,} & \hfill \quad {\frac{T}{4} \le (t - mT) \le \frac{3T}{4}} \\ \hfill {\frac{4A}{T}t - 4A,} & \hfill \quad {\frac{3T}{4} \le (t - mT) \le T} \\ \end{array} } \right.$$

where *m* is the number of cycles (integers 0–59). The formula for *s*_4_(*t*) is presented as follows:

11 $$s_{4} (t) = \left\{ {\begin{array}{*{20}r} \hfill {\frac{4A}{T}t + \theta_{0} ,} & \hfill \quad {0 \le (t - mT) \le \frac{T}{4}} \\ \hfill { - \frac{4A}{T}t + 2A + \theta_{0} ,} & \hfill \quad {\frac{T}{4} \le (t - mT) \le \frac{3T}{4}} \\ \hfill {\frac{4A}{T}t - 4A + \theta_{0} ,} & \hfill \quad {\frac{3T}{4} \le (t - mT) \le T} \\ \end{array} } \right.$$

where *θ*_0_ is the initial phase. In the current study, *θ*_0_ = 20°, 30°, 50°, 90°, 140°, and 170° [3, 9, 13] were tested.

#### Additive noise processes

Noises present in respiratory signals (e.g., electronic and sensor noises) measured using RIP (i.e., RIP_TWM_ and RIP_AWM_) are nondeterministic; therefore, noise should be modeled as stochastic processes. Hence, in line with [15], a Gaussian white process with standard deviation 0.25 was used to model electronic and sensor noises, whereas the high amplitude with low-frequency noise was used to model body movement artifacts. These generated noises were added to the signals to simulate the noise corruptions on these signals. Simulated TWM and AWM signals with correlated noises (i.e., *n*_1_(*t*) = *n*_2_(*t*)) and uncorrelated noises (i.e., *n*_1_(*t*) ≠ *n*_2_(*t*)) were tested.

12 $$n_{1} \left( t \right) = \sigma_{1} \left( t \right) + w_{1} (t)$$

13 $$n_{2} \left( t \right) = \sigma_{2} \left( t \right) + w_{2} (t)$$

Let *w*_1_(*t*) and *w*_2_(*t*) be the independent Gaussian white noises, and let *σ*_*1*_(*t*) and *σ*_2_(*t*) be the high-amplitude with low-frequency noise that corrupts the TWM and AWM signals, respectively.

14 $$\sigma_{1} \left( t \right) = {\text{Asin}}\left( {\frac{2\pi }{T}t} \right)$$

15 $$\sigma_{2} \left( t \right) = {\text{Asin}}\left( {\frac{2\pi }{T}t + \theta_{0} } \right)$$

where A is the amplitude (A = 2), *T* is the cycle period (*T* = 75 s), and *θ*_0_ is the initial phase (*θ*_0_ = 180°) of the low-frequency noise.

### Experiment on human subjects

#### Ethics statement

The data used in this section was from the research project “Paced-respiratory induced heart rate variability and cardiac output evaluation (Protocol No: 100-015-E),” approved by the Institution Review Board of the National Taiwan University Hospital Hsinchu Branch. The committee was organized under and operated in accordance with the Good Clinical Practice guidelines and governmental laws and regulations. Written Informed consents were obtained from all subjects before the experiment. The experiment and the use of the data obtained from human subject were performed in accordance with the approved protocol.

#### *Subjects*

There were 50 subjects that were college students selected from a university in Taiwan, ranging in age between 20 and 23 (21 ± 1 years; 42 men, 8 women), body height between 157.5 and 188 cm (172.05 ± 6.68 cm), body weight between 43 and 87 kg (64.61 ± 9.77 kg), body mass index (BMI) between 15.21 and 29.05 kg/m^2^ (21.77 ± 2.69 kg/m^2^), thoracic circumference between 76 and 105 cm (88.08 ± 6.27 cm), and abdominal circumference between 63.50 and 89 cm (77.15 ± 7.52 cm), were asked to perform TB and AB during the experiment. The subjects were all instructed not to take alcoholic, caffeine-containing drinks or big meals 4 h before the experiment.

#### Experimental procedure

The experiment was carried out in a small, quiet room (7.60 × 3.20 m) without people. The subject wore two RIP sensors (RIPmate Adult Thorax Alice 5 Inductance Kit, Ambu Inc., Denmark) and remained in a seated position in an office chair (0.50 × 0.51 m, height 0.43 m) during the experiment, and was instructed to perform TB and AB. The RIP_TWM_ sensor was worn below the axilla to record TWM, and the RIP_AWM_ sensor was placed on the navel to record AWM. Both RIP signals were acquired by a data acquisition hub (NI SCB-68, National Instruments, USA) and a data acquisition card (NI USB 6255, National Instruments, USA) at a sampling rate of 50 Hz and were subsequently transferred to a computer (Acer Veriton M2610; processor: Intel Core i3-2120 3.3G/3M/65W; memory: 4 GB DDR3-1066; operating system: Microsoft Windows 7 Professional 64 bit). The procedure of the experiment was as follows:

1. The experiment started with an instruction (“Please follow the metronome in the monitor to breath with thoracic breathing/abdominal breathing”) presented on the computer screen (ViewSonic VE700, 17-in, 1280 × 1024-in resolution) for 5 s. The subject then performed TB at a breathing rate (controlled by a metronome on the screen) of 12 breaths per minute (i.e., 0.2 Hz [34–36]) for 5 min. Because TB is the normal breathing type for humans, the subject was asked to breathe normally and focus on the movement of the chest.
2. The subject was instructed to learn AB by using a respiratory signal monitoring system (same as that used in [21]). The system taught the subject AB through three steps.
  - The system instructed the subject to place the left hand on the chest and place the right hand on the abdomen. In addition, the system instructed the subject to focus on the elevation and degradation of the AWM in the second step.
  - The system instructed the subject to inhale deeply through the nose and hold the breath for 2 s, and then exhale through the mouth until the end of the expiration.
  - The system then instructed the subject to repeat the second step continuously for 10 min.
3. After the training session, the subject performed AB at a breathing rate of 12 breaths per minute for an additional 5 min.

The RIP data were recorded during both the TB and AB periods. The program controlling the instructions and data acquisition was developed using LabVIEW platform (LabVIEW 2012, National Instruments, USA).

### Data analysis and comparison

The result obtained using the proposed IPD method was compared with that derived using the improved version of loop analysis [21] and the APEP described in [15]. The loop analysis procedure is described as follows:

1. The method first extracts the TWM and AWM signals from the RIP_TWM_ and RIP_AWM_ as their main components (i.e., the IMF with the highest energy among all the IMFs decomposed from the original signal [28]), respectively.
2. The breathing cycles of the TWM and AWM signals are identified automatically by detecting the valleys of the signal by using a computer program.
3. Loop analysis [3] is applied to the extracted TWM and AWM signals. The relation of two time-dependent functions x(t) and y(t) is displayed in an x–y plot. For each breathing cycle, a Lissajous figure of the rib cage versus abdomen motion signals is drawn. The phase difference (θ) is then calculated using the following equation: sin*(θ)* = *m/s*, where *m* is the length of abdomen motion excursion, which is parallel to the abscissa in the Lissajous figure in the mid-rib cage motion, and *s* is the length of the overall abdomen motion excursion.

The APEP procedure is described as follows [15]:

1. The TWM and AWM signals are submitted into a zero-phase finite-impulse response band-pass filter with parameters set to a high-pass frequency of 0.4 Hz, a high-stop frequency of 0.45 Hz, a low-pass frequency of 0.15 Hz, and a low-stop frequency of 0.1 Hz.
2. The resulting signals are converted using a binary converter that operates pointwise on its input signal, $\tilde{s}\left[ n \right]$, as follows:
  16 $$\tilde{s}\left[ n \right] = \left\{ {\begin{array}{*{20}c} {0, \quad if \tilde{s}\left[ n \right] < 0} \\ {1, \quad if \tilde{s}\left[ n \right] > 0} \\ \end{array} } \right.$$
3. The two resulting signals are submitted into an exclusive-OR gate that operates pointwise on its input signals.
4. The phase difference (θ) of the resulting signal is estimated using a low-pass filter that operates pointwise on its input signal, u[*n*], as follows:
  17 $$\theta [n] = \frac{1}{L}\mathop \sum \limits_{k = 0}^{L - 1} u[n - k]$$

where *L* denotes the number of sample points in the 5-s-long sliding window (*L* = 251).

Bland–Altman analysis was applied to the human-subject data to examine the relation between the phase difference estimation methods [37, 38]. The two-step procedure was carried out according to the recommendations of [39]. First, the correlation between the estimated values (i.e., the equation of the linear relationship and correlation coefficient) was investigated. Second, the relative differences between each pair of measures were plotted against the mean of the pair to determine whether most of the pairs fall within the 95 % limit of the agreement bound.

All numerical analyses in this study were performed using LabVIEW 2012 (National Instruments, USA). Both simulated and human-subject data were sampled at 50 Hz. The significance level of the entire statistical hypothesis tests used in this study, if not stated otherwise herein, was set to 0.05. All statistical analyses were performed using commercial statistics software (Statistical Package for Social Science, Version 22, SPSS Inc., USA).

## Results

### Experiment on simulated time series

Figure 2 shows the 60-s sample results of the phase difference estimations on two triangular signals s_3_(t) and s_4_(t) with correlated (left part) noise (denoted by n_1_(t)) and uncorrelated (right part) noise (denoted by n_1_(t) and n_2_(t)) obtained using the IPD method, loop analysis, and APEP. The frequency of s_3_(t) and s_4_(t) was set to 0.2 Hz, and the phase difference *θ*_0_ was set to 20°. The dashed line represents the gold standard (i.e., *θ*_0_ = 20^∘^). Figure 2 indicates that the average value of the IPD result is closer to *θ*_0_ compared with the average value of loop analysis and APEP results (i.e., 19°, 15° and 19° for IPD, loop analysis, and APEP with correlated noise, respectively; and 20°, 17° and 30° for IPD, loop analysis, and APEP with uncorrelated noise, respectively). This figure also demonstrates that loop analysis generates only one output value per cycle of breathing at the end of each cycle (i.e., 11 output values in the 60-s sample result), whereas both the IPD method and APEP provide pointwise output values (i.e., 60 s × 50 samples/s = 300 samples). The ratio of the number of output values between loop analysis and each of the other two methods is 11:300.

This difference indicates a higher temporal resolution of the phase estimation achieved using the IPD method and APEP, compared with the temporal resolution reached using loop analysis. Table 1 indicates that the IPD method was accurate in all the simulation setups. The results of each of the simulation setups were subjected to one-sample *t* tests to determine whether significant differences existed between the gold standard $\theta_{0}$ and the phase difference estimation results obtained using all the methods. For all the degree levels tested and types of signals used, the absolute differences between the gold standard $\theta_{0}$ and mean value of the IPD results were 0°. The standard deviations of the IPD results were between 0.13° and 0.49°. No significant difference between *θ*_0_ and the IPD estimated phase difference was found in any of the 24 simulation setups. However, the absolute differences between the gold standard $\theta_{0}$ and mean value of the loop analysis results were between 0° and 8°. The standard deviations of the loop analysis results were between 0.1° and 1.04°. Significant differences between *θ*_0_ and the phase difference estimated in loop analysis were found in 21 of the 24 simulation setups. Finally, the absolute differences between the gold standard $\theta_{0}$ and mean value of the APEP results were between 0° and 20°. The standard deviations of the APEP results were between 0.26° and 0.87°. Significant differences between *θ*_0_ and the phase difference estimated in APEP were determined in 10 of the 24 simulation setups. For half of the ten simulation setups, the *θ*_0_ value was larger than 140°. The results indicated an inaccuracy of the APEP in phase degree *θ*_0_ larger than 140°, regardless of the signal or noise types. The absolute differences between the gold standard $\theta_{0}$ and the mean values of the loop analysis results for the triangular signals with correlated noise and uncorrelated noise were higher than the absolute differences for the sinusoidal signals with correlated noise in 10 of the 12 simulation setup. However, no similar tendency was observed in the IPD results or APED results.

**Table 1:** Results of phase difference estimation on various simulated signals

*θ*_0_	Sinusoidal signals with correlated noise			Triangular signals with correlated noise		
	IPD	Loop analysis	APEP	IPD	Loop analysis	APEP
20°	20 ± 0.14	19 ± 0.10^***^	20 ± 0.28	20 ± 0.13	15 ± 0.14^***^	20 ± 0.37
30°	30 ± 0.22	29 ± 0.17^***^	30 ± 0.31	30 ± 0.23	23 ± 0.19^***^	30 ± 0.26
50°	50 ± 0.33	49 ± 0.29^***^	50 ± 0.70	50 ± 0.27	42 ± 0.33^***^	50 ± 0.63
90°	90 ± 0.40	90 ± 0.97	90 ± 0.51	90 ± 0.36	90 ± 1.04^*^	90 ± 0.60
140°	140 ± 0.44	140 ± 0.63^*^	140 ± 0.44^***^	140 ± 0.34	146 ± 0.63^***^	138 ± 0.46^***^
170°	170 ± 0.49	170 ± 0.63^***^	156 ± 0.45^***^	170 ± 0.49	172 ± 0.63^***^	150 ± 0.57^***^

*θ*_0_	Sinusoidal signals with uncorrelated noise			Triangular signals with uncorrelated noise		
	IPD	Loop analysis	APEP	IPD	Loop analysis	APEP
20°	20 ± 0.33	19 ± 0.46^***^	27 ± 0.41^***^	20 ± 0.34	15 ± 0.40^***^	32 ± 0.35^***^
30°	30 ± 0.34	29 ± 0.49^***^	32 ± 0.41^***^	30 ± 0.45	23 ± 0.45^***^	36 ± 0.49^***^
50°	50 ± 0.42	49 ± 0.53^***^	50 ± 0.69	50 ± 0.36	42 ± 0.60^***^	50 ± 0.87^**^
90°	90 ± 0.34	90 ± 0.71	90 ± 0.44	90 ± 0.35	90 ± 0.95	90 ± 0.44
140°	140 ± 0.30	140 ± 0.43^***^	140 ± 0.43	140 ± 0.31	146 ± 0.48^***^	140 ± 0.45
170°	170 ± 0.35	169 ± 0.32^***^	170 ± 0.32	170 ± 0.35	172 ± 0.33^***^	170 ± 0.34^***^

The results of phase difference estimation done by using IPD, loop analysis, and APEP on simulated time series under different simulation setups. The *θ*_0_ and the estimated phase difference were submitted to one-sample *t* test

The significant difference between the estimated phase difference and the gold standard *θ*_0_ is denoted by * p < 0.05, ** p < 0.01, *** p < 0.001

### Experiment on human-subject

**Fig. 3:**
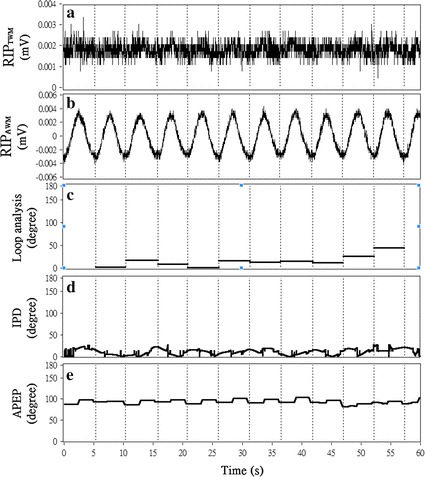
Sample result of the phase difference estimation on human-subject AB data. A 60-s sample result of phase difference estimations on human-subject AB data measured by the RIP_TWM_ (**a**) and RIP_AWM_ (**b**) sensors. The estimation was done by using the loop analysis (**c**), IPD method (**d**), and APEP (**e**). The absent of data for loop analysis in 0–5 and 58–60 s was caused by the incompleteness of cycles. The loop analysis in fact only generates one output value per cycle of breathing at the end of each cycle. The *straight lines* drawn in the* panel* **c** throughout the beginning to the end of each cycle are manually produced after the analysis

In total, two types of breathing (i.e., TB and AB) × 2 (RIP_TWM_ and RIP_AWM_ signal) × 50 (subjects) = 200 rows of the raw data were collected during the experiment. The RIP_TWM_ and RIP_AWM_ signals were decomposed into IMFs by using the CEEMD method described in the “Methods” section. According to the procedure described in the “Methods” section, the sixth IMFs of both the RIP_TWM_ and RIP_AWM_ signals were determined to be the main component (i.e., TWM) of the RIP_TWM_ signal and the main component (i.e., AWM) of the RIP_AWM_ signal, respectively. The frequencies of the extracted TWM signals in TB and the AWM signals in AB were nearly 0.2 Hz. Sample results of the phase difference analysis obtained using our method for IPD estimation, loop analysis, and APEP are presented in Fig. 3. These results indicate that for the IPD method and APEP, the temporal resolution of the result is higher. In other words, the IPD and APEP provided the phase difference estimation at a sampling rate of 50 Hz (i.e., there were 60 s × 50 samples/s = 300 samples for both the IPD and APEP, as shown in Fig. 3), whereas the result of loop analysis was only cycle based (i.e., less than 0.5 Hz; there were 10 output values for loop analysis, as shown in Fig. 3). In addition, it appears that all three methods could still estimate the phase difference between the RIP_TWM_ signal and the RIP_AWM_ signal even when the RIP_TWM_ signal is markedly lower in amplitude (i.e., 0.001 mV; first panel of Fig. 3) than the RIP_AWM_ signal (i.e., 0.004 mV; second panel of Fig. 3).

**Fig. 4:**
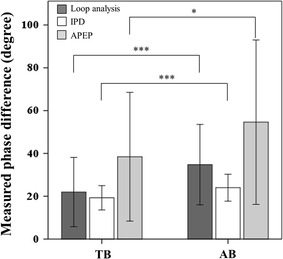
Results of the loop analysis, PD, and APEP on TB and AB data. The result of the phase estimation done by using IPD method, loop analysis, and APEP on the real data. The real data was collected in a 50-subject experiment which asked the subjects to perform TB and AB. The y axis represents the phase difference measured. The significant difference of the phase difference estimated between TB and AB conditions is denoted by *p < 0.05, **p < 0.01, ***p < 0.001

**Fig. 5:**
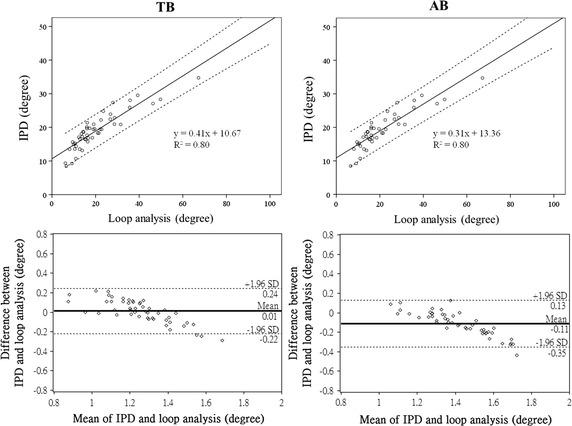
Scatterplots and Bland–Altman analysis result of loop analysis and the proposed IPD method. The *scatterplots* between the phase difference estimated by using loop analysis and IPD for real TB (see the *left side*) and AB (see the *right side*) data are shown in the *upper panel*. The Bland–Altman analysis results of the two methods are shown in the *lower panel*. The *thick lines* in the *lower panel* represent the mean differences of all the paired samples and the *black dotted lines* represent 95 % limits of agreement bound

**Fig. 6:**
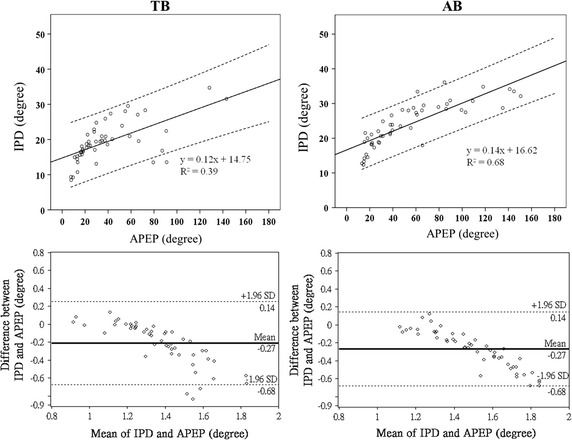
Scatterplots and Bland–Altman analysis result of APEP and the proposed IPD method. The scatterplots between the phase difference estimated by using APEP and IPD for real TB (see the *left side*) and AB (see the *right side*) data are shown in the *upper panel*. The Bland–Altman analysis results of the two methods are shown in the *lower panel*. The *thick lines* in the *lower panel* represent the mean differences of all the paired samples and the *black dotted lines* represent 95 % limits of agreement bound

Figure 4 presents the results of all three phase analysis methods. The paired-sample *t* test results indicate that the estimated phase difference between RIP_TWM_ and RIP_AWM_ signals in AB was significantly greater than that in TB, for the estimation conducted through loop analysis (35 ± 18.77° vs. 22 ± 16.16°, p < .001), the proposed IPD method (24 ± 6.29° vs. 19 ± 5.68°, p < .001), and the APEP (55 ± 38.40° vs. 38 ± 30.08°, p < .05). The correlation analysis results between the IPD method and the other two methods are provided in the upper panels of Figs. 5 and 6 whereas the Bland–Altman analysis results between the IPD method and the other two methods are provided in the lower panels of Figs. 5 and 6.

The data used for Bland–Altman analysis were logarithmically transformed from the original data to ensure a normal distribution, according to the suggestions provided in [38]. Both the result of loop analysis (R^2^ = 0.80 under TB and R^2^ = 0.80 under AB) and the result of APEP (R^2^ = 0.39 under TB and R^2^ = 0.68 under AB) were positively correlated with the result of the proposed IPD method. The Bland–Altman analysis, which examines whether data points that represent the difference between IPD and another method fall within the means ± 2 × standard deviation, revealed that 94 % of the data points in TB and 98 % of the data points in AB for IPD versus loop analysis fell within the means ± 2 × standard deviation. By contrast, 94 % of the data points in TB and all the points in AB for IPD versus APEP fell within the means ± 2 × standard deviation.

## Discussion

### Experiment on simulated time series

Sinusoidal signals are usually used to guide subjects’ inhaled/exhaled volume in the literature [40, 41] because the sinusoidal pattern appears to be similar to respiratory movements of normal subjects [40]. However, respiratory movements are sometimes more triangular in shape than sinusoidal, for instance, in lightly anesthetized rhesus monkeys [23]. In reality, true breathing is neither always triangular nor always sinusoidal. The pattern may be affected by the breathing condition or the disease of a subject. Hence, both sinusoidal wave and triangular wave were used in our simulation. Moreover, according to our simulation result, it is suggested to use the IPD method or the APEP than loop analysis when the target respiratory signal is more triangular than sinusoidal.

The loop analysis was a conventional approach that is proved to be useful and thus widely used in the literature for estimating phase difference. However, the results of the simulated time series show that loop analysis, because of its limitation, is low in temporal resolution. Results of the proposed IPD method and APEP indicate a higher temporal resolution than that of loop analysis. The inaccuracy of loop analysis for the triangular signals is in line with the findings of previous studies [15, 23]. The IPD method and APEP were determined to be more accurate than loop analysis regarding the absolute differences between the gold standard *θ*_0_ and the mean of the estimated values in most of the simulation setups. However, the finding of the inaccuracy of the APEP in estimating *θ*_0_ larger than 140° suggests that the IPD may be a more favorable choice regarding methods for detecting a high degree of phase difference. The estimation of high degrees of phase difference (e.g., from 150° to 179°) is vital because they were found to be highly correlated with certain types of respiratory diseases [3, 9, 13]. Our finding suggests that the competitive performance of the proposed IPD method in estimating *θ*_0_ of sinusoidal and triangular signals was corrupted with correlated/uncorrelated noises, because for all simulation setups, no significant difference was found between the gold standard *θ*_0_ and IPD result. Furthermore, the absolute differences between the gold standard $\theta_{0}$ and mean value of the IPD results were small, and the standard deviations of the IPD results were small. These results indicate the accuracy and the consistency of the proposed method.

**Fig. 7:**
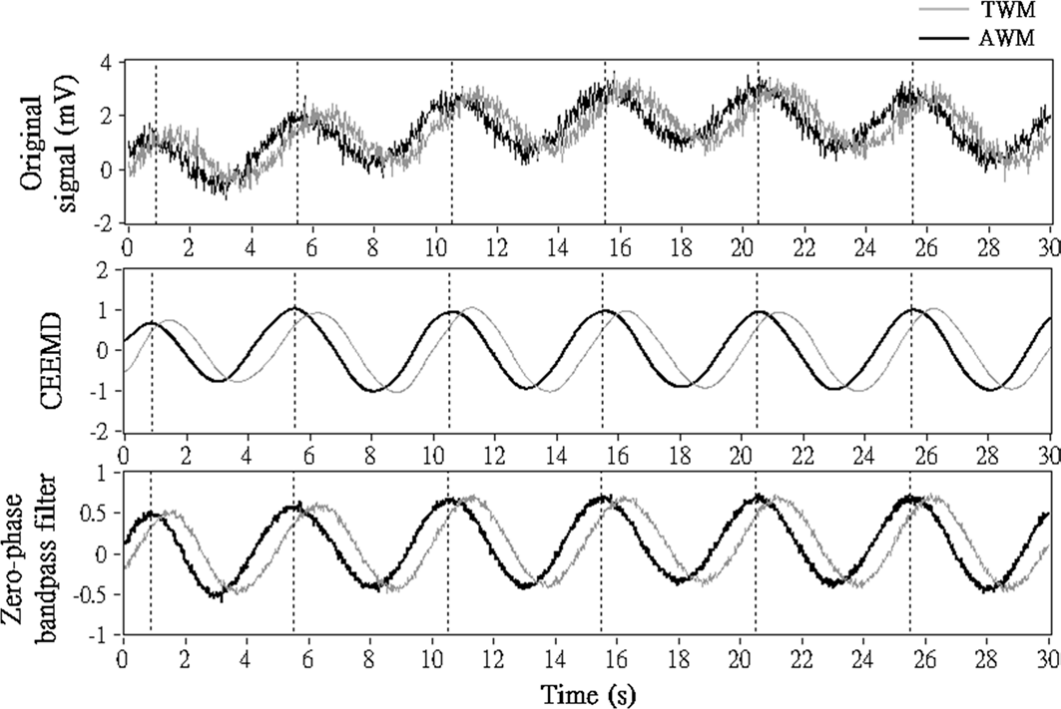
The phase delay of the simulated signals, introduced by the linear-phase band-pass filter. A demonstration of the phase delay of two sinusoidal signals with correlated noise and θ_0_ = 50° (see “Original signal”), introduced by a linear-phase band-pass filter. Please see the 1st panel for the two sinusoidal signals with correlated noise, the 2nd panel for the CEEMD result, and the 3rd panel for the result of the band-pass filter

The magnitude of the absolute difference found between the gold standard *θ*_0_ and the estimation results observed in the simulation is essential and required to be taken into consideration when developing clinical applications. It is because the magnitude of this difference could be proportional to the prediction error in medical diagnosis. This magnitude is not negligible even when it is only 1°, considering this 1° error may affect the analysis result largely in some applications. For instance, the difference in phase angles found in normal subjects and moderate chronic obstructive pulmonary disease patients was just 3.5° [4]. In this case, it is hard to determine the patients with moderate chronic obstructive pulmonary disease using phase angle. Figure 7 demonstrates the effects of CEEMD and a zero-phase band-pass filter on two sinusoidal signals with correlated noises and phase difference *θ*_0_ = 50°. Both the result of the CEEMD filter and the zero-phase band-pass filter appear to be similar to the original signals and have no phase delay to the original signals. Moreover, we also found that CEEMD filter appears to be lesser noisy than the zero-phase band-pass filter in Fig. 7. In addition, despite the fact that the zero-phase filter can be implemented for the APEP method to prevent phase delay, the filter must have time samples available before to start processing. This could be a limitation when developing real-time applications [42].

### Experiment on human-subject

Because the subjects were requested to perform paced breathing at a breathing rate of 12 breaths per minute (i.e., 0.2 Hz), the 0.2-Hz frequency of the CEEMD-extracted TWM signal in TB and the AWM signals in AB seems to be correct. In line with the finding reported by previous research [5–7, 10], all three methods estimated a greater phase difference in AB than in TB (Fig. 4). This finding suggests that the proposed IPD in estimating phase difference in real respiratory data shows a pattern similar to that from using the other two methods. The Bland–Altman and correlation analysis results also indicate that the estimation result of the proposed IPD method shares the same trend with those of the other two methods regarding real respiratory data.

However, the phase difference results estimated through loop analysis (i.e., 22 ± 16.16° for TB and 35 ± 18.77° for AB) and the IPD method (i.e., 19 ± 5.68° for TB and 24 ± 6.29° for AB) are in line with previous findings, in that the phase difference of TWM and AWM under the TB and AB conditions fell within 0° to 40° for healthy subjects [4–9]. This renders the result of the APEP (i.e., 38 ± 30.08° for TB and 55 ± 38.40° for AB) questionable. Furthermore, the IPD had the smallest standard deviation (i.e., 5.68 for TB and 6.29 for AB) among the three methods, whereas the APEP had the largest standard deviation (i.e., 30.08 for TB and 38.40 for AB). This implies that the IPD is a more stable approach to estimating IPD compared with the other two methods.

The correlation analysis results shown in the upper panels of Figs. 5 and 6 present positive correlations between IPD and the other two methods; the R^2^ between the IPD method and APEP (R^2^ = 0.39) was lower than the R^2^ between the IPD method and loop analysis (R^2^ = 0.80) in TB. The large standard deviation (30.08° for TB and 38.40° for AB) and the unexpectedly large phase difference (38° for TB and 55° for AB) of the APEP result (compare with those in the literature [4–9]) may be the reason that the IPD result is correlated with loop analysis result but less correlated with the APEP result. Furthermore, although the IPD result is less correlated with the APEP result compared with that of loop analysis, the IPD results from Bland–Altman analysis were still favorable (lower panels of Figs. 5, 6). Most of the data points of the IPD result fell within the means ± 2 × standard deviation of the loop analysis result and the APEP result. This means that for the real data, the pattern of the phase estimated using the IPD method is similar to those of the other two methods. The negative correlations are depicted in the lower panels of Figs. 5 and 6. In this data set, the exact value of the phase difference estimated using IPD tends to be higher than those estimated using the other two methods when all three methods yield a relatively low phase difference. By contrast, when all three methods yield a relatively high phase difference, the IPD tends to yield lower values than those estimated through loop analysis and the APEP. This leaves room for further research because there is currently no ground truth of the “phase difference”, a quantity that cannot be measured directly by using any type of device. The only current certainty is that the result generated by the IPD method shares a similar tendency with those of the other two methods for real data.

### Research limitations

Despite the experimental results appeared to be promising, the proposed IPD method has certain limitations. First, the use of EMD-based data processing in our method introduces a certain amount of complexity of using the method (same as the recent version of Lissajous figure analysis method proposed in [21]). The EMD-based data processing may sometimes require a researcher to manually determine multiple parameters (e.g., parameters related to the stopping criteria of the iterative decomposition processes) to avoid mode mixing problem and the boundary effect problem [27]. However, in ordinary these parameters can be intuitively determined based on the decomposition result. Another limitation is that the iterative process of the EMD-based data processing may be time consuming [43]. Notably, the order of the computational complexity of the EMD has been shown to be equivalent to FFT, and can be optimized in order to operate in real-time applications [43, 44]. For applications and data analysis tasks operate offline, parallelization [45] and the use of graphics processing unit [46] can further improve the speed of data processing.

## Conclusions

Because thoracoabdominal asynchrony is often used to discriminate respiratory diseases in clinics, the current study presents a novel method (called IPD estimation) based on CEEMD for estimating the phase relation between TWM and AWM signals measured using RIP sensors. Both simulation and human-subject experimental respiratory data show that the proposed IPD estimation method demonstrated competitive performance compared with the recent version of Lissajous figure analysis and the APEP. We believe that the findings of the current study can provide future studies with a new phase difference estimation method that (1) has high temporal resolution, (2) does not introduce phase delay, (3) works with non-sinusoidal signals, (4) provides quantitative phase estimates without estimating the embedded frequency of the breathing signals, and (5) works without calibrated measurements. Thus, the accuracy of the research result may be enhanced. Furthermore, according to the higher temporal resolution of the estimation of phase difference, more information related to the interaction between TWM and AWM can be revealed. We believe that IPD estimation with higher temporal resolution could be a useful aid to the exploration of how the breathing pattern modulates cardiovascular system and autonomic nervous system in the near future.

## Abbreviations

AB: abdominal breathing
AWM: abdominal wall movement
APEP: automatic phase estimation procedure
CEEMD: complementary ensemble empirical mode decomposition
EMD: empirical mode decomposition
IMFs: intrinsic mode functions
IPD: instantaneous phases difference
RIP: respiratory inductance plethysmography
TB: thoracic breathing
TWM: thoracic wall movement
